# Update 2016–2018 of the Nationwide Danish Fungaemia Surveillance Study: Epidemiologic Changes in a 15-Year Perspective

**DOI:** 10.3390/jof7060491

**Published:** 2021-06-19

**Authors:** Malene Risum, Karen Astvad, Helle Krogh Johansen, Henrik Carl Schønheyder, Flemming Rosenvinge, Jenny Dahl Knudsen, Rasmus Krøger Hare, Raluca Datcu, Bent Løwe Røder, Valeria Stanislavovna Antsupova, Lise Kristensen, Jan Berg Gertsen, Jens Kjølseth Møller, Esad Dzajic, Turid Snekloth Søndergaard, Maiken Cavling Arendrup

**Affiliations:** 1 Unit of Mycology, Statens Serum Institut, 2300 København, Denmark; mlri@ssi.dk (M.R.); kaas@ssi.dk (K.A.); rmj@ssi.dk (R.K.H.); Raluca.Datcu@rsyd.dk (R.D.); 2 Department of Clinical Microbiology, University Hospital of Copenhagen, Rigshospitalet, 2100 København, Denmark; hkj@biosustain.dtu.dk (H.K.J.); Inge.Jenny.Dahl.Knudsen@regionh.dk (J.D.K.); 3 Department of Clinical Medicine, University of Copenhagen, 1165 København, Denmark; 4 Department of Clinical Microbiology, Aalborg University Hospital, 9100 Aalborg, Denmark; henschoenh@gmail.com; 5 Department of Clinical Medicine, Aalborg University, 9220 Aalborg, Denmark; 6 Department of Clinical Microbiology, Odense University Hospital, 5000 Odense, Denmark; Flemming.rosenvinge@rsyd.dk; 7 Department of Clinical Microbiology, Hvidovre Hospital, 2650 Hvidovre, Denmark; 8 Department of Clinical Microbiology, Slagelse Sygehus, 4200 Slagelse, Denmark; blro@regionsjaelland.dk; 9 Department of Clinical Microbiology, Herlev and Gentofte Hospital, 2730 Herlev, Denmark; valeria.stanislavovna.antsupova@regionh.dk; 10 Department of Clinical Microbiology, Aarhus University Hospital, 8200 Aarhus, Denmark; lise.kristensen@auh.rm.dk (L.K.); Jan.Berg.Gertsen@rm.dk (J.B.G.); 11 Department of Clinical Microbiology, Vejle Sygehus, University Hospital of Southern Denmark, 7100 Vejle, Denmark; Jens.Kjoelseth.Moeller@rsyd.dk; 12 Department of Clinical Microbiology, Sydvestjysk Sygehus, 6700 Esbjerg, Denmark; Esad.Dzajic@rsyd.dk; 13 Department of Clinical Microbiology, Sygehus Sønderjylland, 6400 Sønderborg, Denmark; Turid.Snekloth.Sondergaard@rsyd.dk

**Keywords:** *Candida*, candidaemia, candidiasis, resistance, echinocandin, mutation, epidemiology

## Abstract

As part of a national surveillance programme initiated in 2004, fungal blood isolates from 2016–2018 underwent species identification and EUCAST susceptibility testing. The epidemiology was described and compared to data from previous years. In 2016–2018, 1454 unique isolates were included. The fungaemia rate was 8.13/100,000 inhabitants compared to 8.64, 9.03, and 8.38 in 2004–2007, 2008–2011, and 2012–2015, respectively. Half of the cases (52.8%) involved patients 60–79 years old and the incidence was highest in males ≥70 years old. *Candida albicans* accounted for 42.1% of all isolates and *Candida glabrata* for 32.1%. *C. albicans* was more frequent in males (*p* = 0.03) and *C. glabrata* in females (*p* = 0.03). During the four periods, the proportion of *C. albicans* decreased (*p* < 0.001), and *C. glabrata* increased (*p* < 0.001). Consequently, fluconazole susceptibility gradually decreased from 68.5% to 59.0% (*p* < 0.001). Acquired fluconazole resistance was found in 4.6% *Candida* isolates in 2016–2018. Acquired echinocandin resistance increased during the four periods 0.0%, 0.6%, 1.7% to 1.5% (*p* < 0.0001). Sixteen echinocandin-resistant isolates from 2016–2018 harboured well-known *FKS* resistance-mutations and one echinocandin-resistant *C. albicans* had an *FKS* mutation outside the hotspot (P1354P/S) of unknown importance. In *C. glabrata* specifically, echinocandin resistance was detected in 12/460 (2.6%) in 2016–2018 whereas multidrug-class resistance was rare (1/460 isolates (0.2%)). Since the increase in incidence during 2004–2011, the incidence has stabilised. In contrast, the species distribution has changed gradually over the 15 years, with increased *C. glabrata* at the expense of *C. albicans*. The consequent decreased fluconazole susceptibility and the emergence of acquired echinocandin resistance complicates the management of fungaemia and calls for antifungal drug development.

## 1. Introduction

Candidaemia is the most common manifestation of fungaemia and of invasive candidiasis [1]. The overall 30-day mortality rate was 43% and even higher (54%) in the intensive care unit (ICU) in a nationwide study in Denmark in 2010–2011 [2]. Host factors include multimorbidity and gastrointestinal disease [2]. Main risk factors are prior abdominal/complicated surgery, antibiotic exposure, an indwelling central venous catheter, and *Candida* colonisation [2]. The recommended first-line treatment of candidaemia is echinocandin [3,4,5].

The Danish candidaemia surveillance has existed since 2003 [6,7] and has been nationwide since 2004 [8,9,10]. The highest annual incidence in Denmark was 10.05/100,000 inhabitants in 2011 [9]. During the surveillance period echinocandin resistance has emerged and fluconazole non-susceptibility increased [10]. Echinocandin resistance has emerged particularly in *C. glabrata.* The target enzyme for the echinocandins, the β-(1,3)-d-glucan synthase, is encoded by the *FKS* genes [11,12]. Mutations in specific “hotspot” regions of *FKS1* for all *Candida* species as well as *FKS2* for *C. glabrata* lead to MIC elevation and reduce the sensitivity of the enzyme up to several thousandfold [13]. The position of the mutation, the specific amino acid alteration, and the species in which it is inserted all affect the level of resistance [11]. Therefore, *FKS* sequencing is highly informative and essential for interpretation, particularly of MICs close to the echinocandin breakpoint. 

Denmark has the highest fungaemia incidence among the Nordic countries [14,15,16,17,18]. Suggested causes have been a higher antibacterial drug use and a higher prevalence of haematological malignancy [19]. The impact of antibiotic use was supported by a Danish study on ICU patients, which found that exposure to ciprofloxacin-containing antibiotics increased the risk of invasive *Candida* infections [20]. In contrast, the importance of differences in prevalence of underlying haematologic malignancy was not supported, as only a minority of Danish candidaemia patients had underlying haematological disease (9%) [2].

Denmark has also experienced a larger shift in species proportion from *Candida albicans* to *Candida glabrata* than the other Nordic countries and has had the highest consumption of antifungal drugs in both the primary and hospital sector [19]. Prior antifungal use has been shown to lead to a higher proportion of candidaemia with non-*C. albicans* species, especially *C. glabrata* following azole and *C. parapsilosis* following echinocandin exposure [1]. Azole antifungal agents are recommended as prophylaxis for certain patient groups in Denmark depending on their underlying disease and risk factors—especially in the ICU, in haematological patients, in low-birth-weight neonates, and in lung and liver transplant recipients [3]. 

Due to the changing epidemiology, the active nationwide surveillance programme has continued. Knowledge of the local epidemiology is important for timely revision of guidelines for initial antifungal therapy and informative with respect to whether changes in antifungal stewardship approaches, infection control, or prophylaxis strategies are needed. We report the most recent national data on the epidemiology of fungaemia including antifungal susceptibility over a 15-year perspective.

## 2. Materials and Methods

### 2.1. Isolates, Episode Definition, and Blood-Culture Systems

Fungal blood isolates were referred to the National Reference Mycology Laboratory at Statens Serum Institut for species verification and susceptibility testing from the ten Danish clinical microbiological departments in the years 2016 to 2018. Thirteen isolates (0.9%) were not referred for confirmatory identification and susceptibility testing, but are included in the analysis according to the species identification performed locally. These included: *C. glabrata n* = 6, *C. albicans n* = 4, *C. krusei n* = 1, *Candida parapsilosis n* = 1, and *Candida dubliniensis n* = 1. Confirmatory species identification was performed based upon morphology and Matrix-assisted laser desorption/ionisation time-of-flight mass spectrometry (Bruker, Bremen, Germany) [9] with the online available spectrum database MSI [21] or DNA sequencing as previously described when needed [10]. 

Isolates were considered unique despite originating from the same patient if either (a) belonging to a different species, (b) having a different susceptibility pattern, or (c) obtained more than 21 days apart. Non-unique isolates were excluded. An episode was defined as polyfungal when more than one species was isolated in blood cultures obtained the same day. The numbers of episodes were centre-based, in accordance with previous Danish candidaemia publications [7,9,10,22]. The incidences are defined as number of episodes relative to the number of inhabitants, discharges, or bed days. 

Most departments served several hospitals. Four centres used BacT/ALERT (bioMérieux, Marcy l’Etoile, France), one centre used BACTEC (Becton Dickinson, Franklin Lakes, NJ, USA), and the remaining five either changed systems during the study period or used both systems concomitantly (Supplementary Table S1). 

### 2.2. Susceptibility Testing and FKS Gene Sequence Analysis

Susceptibility testing was done prospectively for ≥98.8% of the isolates according to EUCAST E.Def 7.3 [23]. Stock solutions (5000 mg/L in dimethyl sulfoxide (DMSO; Sigma-Aldrich, Brøndby, Denmark) were used of the following compounds: fluconazole, voriconazole and amphotericin B (Sigma- Aldrich), anidulafungin (Pfizer A/S, Ballerup, Denmark), and micafungin (Astellas Pharma Inc., Tokyo, Japan; and Molcan Corporation, Toronto, Canada from 15 May 2018). *C. parapsilosis* ATCC 22019, or *C. krusei* ATCC 6258, or both were included as quality controls. The final test concentration ranges varied over the years, but the following concentrations were included throughout: amphotericin B 0.016–4 mg/L, anidulafungin 0.008–1 mg/L (*C. dubliniensis*: 0.004–1 mg/L), micafungin 0.008–1 mg/L, fluconazole 0.125–16 mg/L, and voriconazole 0.03–4 mg/L. Susceptibility classification was performed adopting the current EUCAST clinical breakpoints v. 10.0 [24]. For drug-species combinations without breakpoints, MICs were interpreted as follows. For amphotericin B, the non-species-specific susceptibility breakpoint of 1 mg/L was used for all species, except *C. lusitaniae* (which is regarded as intrinsically resistant regardless of the MIC due to a high mutation rate and lower amphotericin B cidality [25]). For fluconazole, the EUCAST non-species-specific breakpoints were used (S: ≤2 and R: >4 mg/L) for all *Candida* spp. For echinocandins against *C. dubliniensis* specifically, single-centre 99% wild-type upper limits (WT-UL_99_) were determined using the ECOFF finder program v. 2.1 [26] and adopted as susceptibility breakpoints (anidulafungin: ≤0.03 mg/L and micafungin: ≤0.06 mg/L). Finally, established EUCAST ECOFFs were used to determine the proportion of non-wildtype isolates according to the European Committee on Antimicrobial Susceptibility Testing [27].

*FKS* sequencing was performed as previously described for *Candida* isolates with an elevated echinocandin MIC [10]. In case of discordant susceptibility classification for anidulafungin and micafungin, the isolate was deemed resistant if *FKS* sequencing confirmed a known hotspot alteration. Acquired echinocandin resistance rates were determined for *C. albicans, C. dubliniensis, C. glabrata, C. krusei, Candida tropicalis,* and *Candida kefyr*, and compared with data from the previous years [10].

### 2.3. Population Data

Annual Danish population data from the first quartile was obtained from dst.dk accessed on 25 May 2021. The total numbers of discharges and bed days among somatic admissions were obtained and accessed on 19 December 2019 from www.esundhed.dk. Furthermore, the microbiologists at the clinical microbiological departments provided data separately from their own centre. Numbers of selected abdominal surgical procedures were available at www.esundhed.dk accessed on 25 May 2021, from the period 2005 to 2018. 

In order to use Poisson regression analysis and compare numbers of episodes in patient groups relative to the population number per year, patients count individually per year and according to patient ID in the current study period. 

### 2.4. Consumption of Antifungal Compounds

The antifungal consumption for Denmark was retrieved for primary health care sector and hospital from the website www.medstat.dk, obtained and accessed on 8 July 2020. Global antifungal use for Norway, Sweden, and Finland (DDD/1000 inhabitants/year) was acquired from Grossistbasert legemiddelstatistikk, Folkehelseinstituttet or in English: Norwegian Drug Wholesales Statistics, Norwegian Institute of Public Health—www.fhi.no, obtained and accessed on 21 August 2020; www.socialstyrelsen.se, obtained and accessed on 15 August 2017; and www.firmea.fi, obtained and accessed on 6 September 2020, respectively. Data on antifungal consumption in Sweden were not available for 2017 and 2018, thus the comparison is made only for Denmark, Norway, and Finland for these years. 

### 2.5. Statistics

The ꭓ^2^-test was used when comparing isolate proportions in groups, and Fischer’s exact test was used when the expected counts were <5. The ꭓ^2^-test for a trend was used when comparing isolate proportions in a four-period time interval or more than two age groups, using GraphPad Prism v. 8.3.0. (San Diego, CA, USA). A negative binominal dispersion was used when comparing numbers of episodes relative to the number of bed days in the four periods as well as numbers of episodes relative to the numbers of inhabitants for the period 2011 to 2018 using IBM SPSS Statistics v. 26 (Armonk, New York, NY, USA). A Poisson regression analysis was used for numbers of episodes in gender and age groups using IBM SPSS Statistics v. 26. 

The study was approved by Compliance at Statens Serum Institut (journal number: 21/00993).

## 3. Results

**Figure 1:**
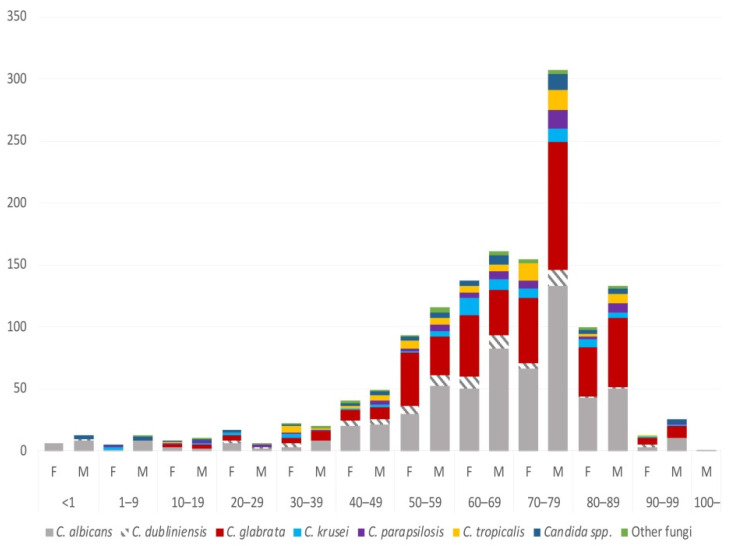
Number of isolates according to age and gender in 2016 to 2018.

A total of 1454 unique blood isolates from 1402 unique episodes in 1311 patients were collected in the years 2016 to 2018 (Table 1). Half of the candidaemia cases (52.8%) involved patients 60–79 years old (Supplementary Table S2). Most episodes occurred in males (60%) (Table 1). The age and gender group that contained the highest number of isolates (21.1%) was males 70–79 years old (Figure 1). 

### 3.1. Incidence

**Figure 2:**
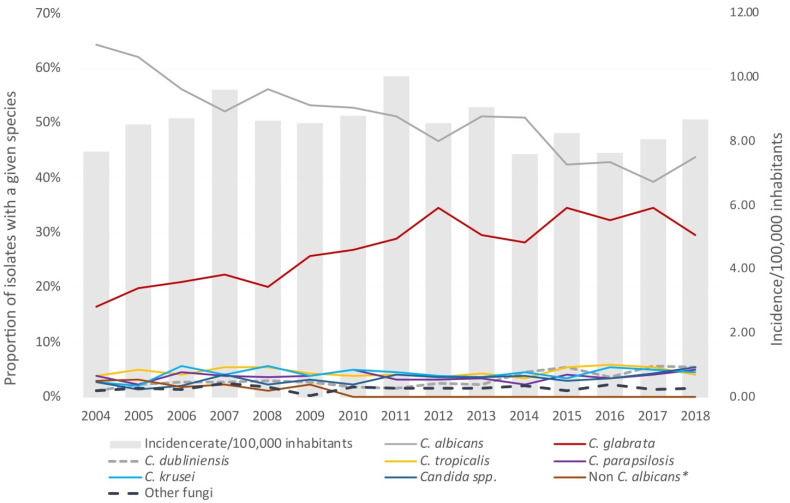
Fungaemia species distribution and incidence per 100,000 inhabitants in 2004 to 2018. * The term non-*C. albicans* was used from 2004 to 2009.

**Figure 3:**
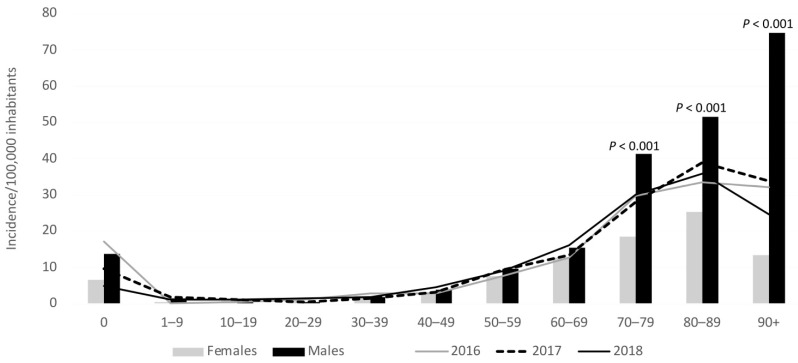
Age- and gender-specific episode rates in 2016–2018 (bars) and age-specific incidences each year (lines). Incidences are determined based on the number of episodes per inhabitants in the specific age and gender groups. Population data for the age group 90+ are based on the number of inhabitants from 90 years up to 109 years old. The *p*-values are stated when statistically significant between genders in the specific age group.

The incidence was 8.13/100,000 inhabitants in 2016–2018 compared to 8.64, 9.03 and 8.38 per 100,000 inhabitants in the time periods 2004–2007, 2008–2011, and 2012–2015, respectively (Table 1, Table 2, and Figure 2). The incidence did not decrease significantly from the peak in 2011 to 2018 (*p* = 0.08). The numbers of episodes relative to the numbers of bed days increased significantly in the four periods (*p* < 0.001). The highest incidences were observed at the extremes of age (Figure 3). Gender-specific incidences were different: there were 9.73/100,000 male inhabitants and 6.56/100,000 female inhabitants in 2016–2018, with a male/female incidence rate ratio (IRR) of 1.666 (95% CI: 1.655–1.668).

The incidence rate was significantly higher in the older age groups for both males (80–89 and ≥90 years) and for females (80–89 years) compared to all other age groups (Figure 3, Supplementary Table S3).

### 3.2. Species Distribution

*C. albicans* (42.1%) and *C. glabrata* (32.1%) were the two dominant species in 2016–2018. *C. albicans* accounted for less than 40% in 2017 (Table 1, Figure 2). *C. tropicalis, C. krusei, C. dubliniensis*, and *C. parapsilosis* each accounted for ≤5.2%; other *Candida* species 4.5%; and isolates (*n* = 27) other than *Candida* accounted for 1.9% (Table 1, Figure 2). Polyfungal episodes (*n* = 52) accounted for 3.7% of the episodes and half of these involved *C. albicans* and *C. glabrata* (*n* = 27, 1.9% of all). Notable differences in the epidemiology were observed between the specific centres (Supplementary Table S1). Comparing the seven centres serving university (± district) hospitals that all had >100 (range 149–258) blood-culture isolates, the incidence varied three-fold (5.62–14.6/100,000 inhabitants and 0.59–1.94/10,000 bed days), and the *C. albicans* and *C. glabrata* proportions varied 34.4–48.4% and 24.7–38.0%, respectively.

During 2004–2018 *C. albicans* decreased (64.4% to 43.7%) and *C. glabrata* increased (16.5% to 29.7%) [22], (both *p* < 0.0001 compared with 2004–2007, 2008–2011, 2012 –2015, and 2016–2018). Moreover, *C. dubliniensis* and other *Candida* species increased (*p* < 0.001 and *p* = 0.004, respectively), whereas the proportions of *C. krusei, C. parapsilosis*, and *C. tropicalis* remained stable (Figure 2).

### 3.3. Species and Gender

In 2016–2018 *C. albicans* was more common in males than females (44.4% and 38.8%, *p* = 0.03), and *C. glabrata* was more common in females than in males (35.3% and 29.9%, *p* = 0.03).

### 3.4. Species and Age

**Figure 4:**
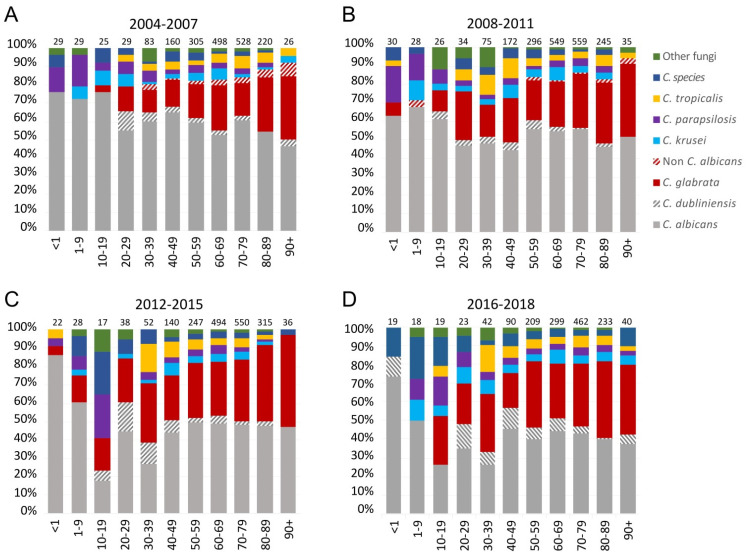
Species distribution in proportions (%) among age groups (years) in the four periods 2004 to 2007 (**A**), 2008 to 2011 (**B**), 2012 to 2015 (**C**), and 2016 to 2018 (**D**). Number of total number of isolates within one age group is stated. Data from 2004 to 2015 have previously been published by Astvad et al. [10].

The proportion of *C. glabrata* isolates increased with increasing age group (*p* < 0.001). Of note, *C. parapsilosis* was not detected among patients <1 years, and only a single *C. parapsilosis* has been found in this age group (2.4%) since 2012 as compared to 10 (16.9%) during 2004–2011 (*p*= 0.03) (Figure 4). The age-specific species distribution also varied among the centres. The referral hospital Rigshospitalet had the lowest proportion of *C. glabrata* and the highest proportion of patients with candidaemia in the age group below 50 years. Details of blood-culture systems and centre-specific incidences are presented in Supplementary Table S1.

### 3.5. Susceptibility

MICs for the 1439 isolates referred for susceptibility testing are shown in Table 3. Thirteen isolates were not referred, and two isolates were not susceptibility tested, as further detailed in the methods section.

Acquired echinocandin resistance was detected in 19 isolates: 12/460 *C. glabrata* isolates, 5/72 *C. krusei* isolates, 1/72 *C. dubliniensis*, 1/608 *C. albicans*, 0/75 *C. tropicalis*, and 0/8 *C. kefyr*. The acquired echinocandin resistance rate among species that are normally susceptible but in which acquired resistance has previously been reported was 1.5% (19/1295) in 2016–2018, and increased in a four-period perspective (*p* <0.001). Of the resistant isolates, 16 harboured *FKS* hot-spot alterations. Six isolates harboured *FKS* alterations outside the hot-spot regions (Table 4). The echinocandin resistance rate in *C. glabrata*, specifically, was 2.6% (12/460). Of those 8.3% (1/12) was also fluconazole resistant, but none displayed resistance to amphotericin B. Susceptibility to amphotericin B was overall 99.9%.

Acquired fluconazole resistance accounted for 4.6% (59/1276) among the most common species that are normally either S or I to fluconazole. In detail, acquired fluconazole resistance was detected in 0.5% (3/608) *C. albicans*, 4.2% (3/72) *C. dubliniensis*, 10.7% (49/460) *C. glabrata*, 1.6% (1/61) *C. parapsilosis*, and 4.0% (3/75) *C. tropicalis*. Combined fluconazole and echinocandin resistance was found in one *C. albicans*, one *C. glabrata*, and five *C. krusei*. Voriconazole resistance/non-wild-type phenotype was detected in 3.7% (47/1276) of the same most common species including 0.7% (4/608) *C. albicans*, 0.0% (0/72) *C. dubliniensis*, 8.3% (38/460) *C. glabrata*, 5.3% (4/75) *C. tropicalis*, and 1.6% (1/61) *C. parapsilosis*. The proportion of fluconazole-susceptible isolates (at standard dosing) decreased to 59.0% (848/1438) in a four-period perspective from 68.5% (972/1420), 65.2% (1137/1745), and 60.6% (1147/1892) in 2004–2007, 2008–2011, and 2012–2015, respectively (*p* < 0.001, Table 2).

### 3.6. Antifungal Consumption

**Figure 5:**
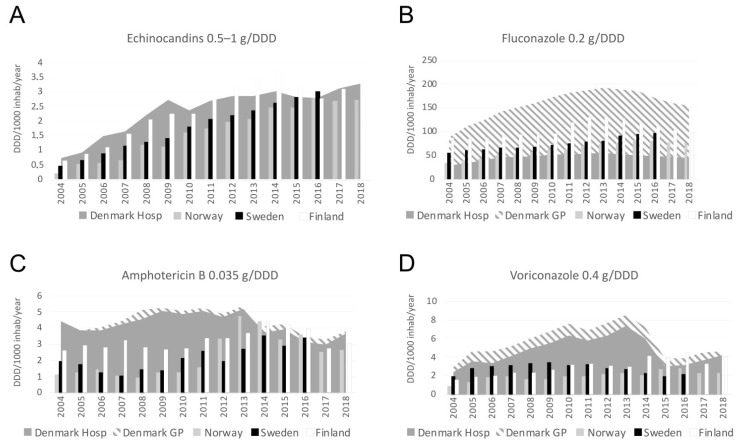
Annual consumption of selected antifungal compounds ((**A**) Echinocandins, (**B**) Fluconazole, (**C**) Amphotericin B, (**D**) voriconazole) shown in DDDs per 1000 inhabitants per year in 2004 to 2018. For Denmark the usage is divided into hospital use (Hosp) and general practitioner use (GP). Data from 2004 to 2015 were also shown in the surveillance by Astvad et al. [10].

The consumption of the antifungal agents amphotericin B, fluconazole, voriconazole, and posaconazole in Denmark peaked in the years 2012 to 2014. The consumption of echinocandins has increased in Danish hospitals since 2004 and was highest in 2018 (3.3 DDDs/1000 inhabitants/year) (Figure 5 and Supplementary Figure S1). The total consumption of fluconazole was highest in Denmark during 2012–2015 and decreased during the current three-year study period to 172, 160, and 155 DDDs/1000 inhabitant/year, respectively (Figure 5). During 2016–2018, 71% (1,988,000/2,803,000 DDD) of the total fluconazole use was prescribed in the primary healthcare sector, including 74% (1,462,000/1,978,000 DDDs) prescribed in women.

From a Nordic perspective, the consumption of echinocandins was comparable to that in the other Nordic countries. The consumption of fluconazole and posaconazole remained notably larger in Denmark than in the other Nordic countries, and the consumption of voriconazole and itraconazole was larger in Denmark than in Sweden and Norway (Figure 5 and Supplementary Figure S1).

## 4. Discussion

We previously reported an increase in incidence up until 2011 and a slight decrease in 2012–2015 [10] but this trend did not seem to continue during 2016–2018. In contrast, the incidence appears to have stabilised. Consequently, Denmark remained a high incidence country from both Nordic and global perspectives, with an incidence similar to the one found in the CDC’s Emerging Infections Program US [14,15,16,17,18,28]. The species distribution, however, continued to shift towards a higher proportion of *C. glabrata* and a lower proportion of *C. albicans* (even below 40% in 2017). A *C. albicans* proportion below 40% has been reported in the US and South America [28,29,30,31,32], but no other Nordic country has reported a *C. albicans* proportion below 50% [14,16,17,33]. This change in species distribution was the main cause of the observed decrease in overall fluconazole susceptibility, yet acquired fluconazole and voriconazole resistances were found in 4.6% and 3.7%, respectively, of the normally susceptible common *Candida* spp. isolates. The echinocandin resistance rate increased in a four-period perspective (where also the echinocandin use increased), but remained stable during 2012–2018 and less common than acquired resistance to fluconazole [10]. *C. glabrata* was confirmed as the species with the highest rates of acquired echinocandin, fluconazole, and voriconazole resistance as also found elsewhere [14,28,34]. Of note, no cases involving *C. auris* were detected in Denmark during the observation period.

Candidaemia remained most frequent in males in accordance with previous findings globally [2,9,10,14,15,16,33,35,36,37,38,39,40,41,42]. The median age (70 years) was slightly higher but nevertheless in accordance with previous studies in Denmark, other Nordic countries (64–69 years) and elsewhere (all above 60 years) [10,14,16,31,33,35,37,38,39,42]. The incidence was highest among males in the age group ≥90 years, and thus later in life than previously [10]. Since the surveillance programme was initiated, life expectancy has increased by 3.6 and 2.7 years for Danish males and females, respectively, which may be part of the explanation for the increase in median age (life expectancy—Statistics Denmark). Moreover, an increasing number of surgical procedures and a minor increase in number of admissions to the ICU in the elderly age groups (www.esundhed.dk/Registre accessed on 31 March 2021 and Regionernes Kliniske Kvalitetsudviklingsprogram from the Danish Intensive Database provided 8 June 2021, respectively) suggest that more intensive management strategies are currently offered to the elderly population.

The proportion of *C. glabrata* isolates increased over time, with age, and was still most common in females [10]. Underlying drivers may be the growing elderly population and a high azole use in Denmark, which remained higher than in the other Nordic countries, and which in the primary health care sector is three times higher in females than in males. *C. parapsilosis* was not detected in children of less than one year of age during the current 3-year surveillance, and number of isolates was found to be significantly less common in comparing the periods 2012–2018 with 2004–2011. This was somewhat surprising as *C. parapsilosis* historically has been the second most common species in this age group in Denmark and elsewhere [6,9,22,43,44]. A recent European paediatric study found geographical differences in incidences of *C. parapsilosis* with the highest incidence in Southern Europe [44]. It is unknown whether these differences over time and between countries are potentially related to differences in infection control practices, use of prophylaxis, or composition of the normal colonising flora.

This study has strengths and limitations. The major strength is that it is population based, nationwide, and includes 15 years of continued surveillance. A limitation is the lack of clinical data and patient-specific antifungal medication. Moreover, differences over time and among centres in blood-culture practices (blood-culture system and sample volume) and antifungal prophylaxis may impact blood-culture sensitivity overall and for the individual species differentially [8]. Another potential caveat is that patients’ episodes are counted twice when transferred between centres and when blood culture is positive at both sites, in order to allow centre-specific incidence comparisons of the candidaemia burden. However, since the initiation of the nationwide surveillance, the number of clinical microbiological departments have been reduced—potentially leading to fewer transfers between centres and thus fewer cases being counted twice. Moreover, the number of transfer cases was limited (19 (1.4%) episodes), resulting in a corrected nationwide incidence of 8.02 rather than 8.13/100,000 inhabitants if transferred cases were omitted, a difference that does not affect the overall findings and conclusions of the study.

In conclusion, we found a stable incidence of fungaemia since the peak in 2011 with a continued shift in species proportion towards *C. glabrata*, a decreasing overall azole susceptibility rate and increase in acquired echinocandin and azole resistance, especially in *C. glabrata*. This leads to challenges in management of candidaemia since echinocandin treatment in some cases is inappropriate and de-escalation to fluconazole less often possible. This highlights the need for antifungal stewardship and new antifungal agents with alternative targets. Notable differences were found in comparing the epidemiology between the centres, illustrating the importance of following the local epidemiology.

## Figures and Tables

**Table 1:** Population and patient demographics, episode rates, and species distribution.

	2016	2017	2018	2016–2018
Number of isolates	453	482	519	1454
Number of episodes	436	464	502	1402
Number of patients	409	432	470	1311
Median age (range)	70 (0–96)	71 (0–102)	69.5 (0–96)	70 (0–102)
Number of episodes in males/females (%)	260/176 (59/41)	267/197 (58/42)	307/195 (62/38)	834/568 (60/40)
Population	5,707,251	5,748,768	5,781,190	5,745,736.3 ^a^
Incidences of episodes				
Per 100,000 inhabitants	7.64	8.07	8.68	8.13
Per 1000 discharges	0.31	0.33	0.37	0.34
Per 10,000 bed days	1.02	1.10	1.21	1.11
Number of isolates (% of total)				
*C. albicans*	195 (43%)	190 (39.4%)	227 (43.7%)	612 (42.1%)
*C. glabrata*	146 (32.2%)	167 (34.6%)	154 (29.7%)	467 (32.1%)
*C. dubliniensis*	17 (3.8%)	27 (5.6%)	29 (5.6%)	73 (5.0%)
*C. tropicalis*	27 (6.0%)	26 (5.4%)	22 (4.2%)	75 (5.2%)
*C. parapsilosis*	16 (3.5%)	20 (4.1%)	26 (5.0%)	62 (4.3%)
*C. krusei*	25 (5.5%)	24 (5.0%)	24 (4.6%)	73 (5.0%)
*Candida* spp. ^b^	16 (3.5%)	21 (4.4%)	28 (5.4%)	65 (4.5%)
Other fungi ^c^	11 (2.4%)	7 (1.5%)	9 (1.7%)	27 (1.9%)

^a^ mean population during the three years. ^b^ Candida spp.: C. lusitanae (n = 22), C. guilliermondii (9), C. kefyr (8), C. orthopsilosis (5), C. metapsilosis (3), C. pelliculosa (3), C. species (3), C. fermentati (2), C. inconspicua (2), C. norvegensis (2), C. utilis (2), C. nivariensis (1), C. pararugosa or Wickerhamiella pararugosa (1), Lodderomyces elongisporus (1), C. eremophila (Pichia kluyveri) (1). ^c^ Other fungi: Cryptococcus neoformans (9), Saccharomyces cerevisiae (7), Magnusiomyces capitatus (formerly known as Geotrichum capitatum) (4), Saccharomyces telluris (1), Cryptococcus albidus (1), Rhodotorula mucilaginosa (1), Fusarium dimerum (1), Fusarium solani (1), Barnettozyma salicaria (formerly known as Pichia salicaria) (1), and mould not further identified due to insufficient growth (1).

**Table 2:** Data in a four-period perspective.

	2004–2007	2008–2011	2012–2015	2016–2018
**Incidences of episodes**				
per 100,000 inhabitants	8.64	9.03	8.38	8.13
per 1000 discharges	0.39	0.38	0.34	0.34
per 10,000 bed days	0.90	1.03	1.06	1.11
**Elderly population (≥70 years)**	573,697	600,248	661,414	761,795
Numbers of selected surgical procedures (mean/year)	134,468	152,086	195,329	238,072
Numbers of admission to the intensive care unit (mean/year)	11,193	11,345	12,472	12,596
**Susceptibility**				
Echinocandin acquired resistance rate (%)	0 (0/1294)	0.6 (10/1581)	1.7 (29/1754)	1.5 (19/1295)
Fluconazole susceptibility rate (%)	68.5 (972/1420)	65.2 (1137/1745)	60.6 (1147/1892)	59.0 (848/1438)

The number for the elderly population ≥70 years is the mean number per year in the stated period. Numbers of selected surgical procedures and bed days were available at www.esundhed.dk accessed on 25 May 2021. Operations on the digestive tract and spleen, punctures and punctual biopsies/smaller surgeries, and endoscopies were chosen. Numbers stated as “<5” at www.esundhed.dk accessed on 25 May 2021 were not included in the calculations. Numbers of admissions to the intensive care unit were provided by RKKP (Regionernes Kliniske Kvalitetsudviklingsprogram) from the Danish Intensive Database.

**Table 3:** Susceptibility table for fungal blood isolates in 2016 to 2018.

Species and Compound			Number of Isolates with the Given MIC (mg/L)											S		R		Non-Wildtype	
	≤0.008	0.016	0.03	0.06	0.125	0.25	0.5	1	2	4	8	16	≥32	Number of Isolates	%	Number of Isolates	%	Number of Isolates	%
***C. albicans*** (*n* = 608)																			
Amphotericin B				19	186	354	49							608	100%	0	0	0	0
Anidulafungin	577	29	2											608	100%	0	0	0	0
Micafungin	362	228	**17**	**1**										607	99.8%	1	0.2%	18	3.0%
Fluconazole					275	303	22	**1**	**1**	**3**			**3**	602	99.0%	3	0.5%	8	1.3%
Voriconazole			602	2				**1**	**1**		**2**			604	99.3%	4	0.7%	6	1.0%
***C. dubliniensis*** (*n* = 72)																			
Amphotericin B		6	28	28	10									72	100%	0	0	0	0
Anidulafungin	37 *	29	5			**1**								71	98.6%	1	1.4%	ND	ND
Micafungin	11	31	27	2					**1**					71	98.6%	1	1.4%	ND	ND
Fluconazole					33	28	7			**1**		**1**	**2**	68	94.4%	3	4.2%	4	5.6%
Voriconazole			69		**2**	**1**								69	95.8%	0	0	3	4.2%
***C. glabrata*** (*n* = 460)																			
Amphotericin B			3	11	58	239	148	1						460	100%	0	0	0	0
Anidulafungin	10	167	212	65	**1**	**2**	**1**	**2**						454	98.7%	6	1.3%	6	1.3%
Micafungin	90	247	113	**5**	**2**		**2**	**1**						450	97.8%	10	2.2%	10	2.2%
Fluconazole							2	10	142	223	28	6	**49**	0	0	49	10.7%	49	10.6%
Voriconazole			53	252	92	9	5	11	**26**	**10**	**2**			IE	IE	IE	IE	38	8.3%
***C. krusei*** (*n* = 72)																			
Amphotericin B						1	47	24						72	100%	0	0	0	0
Anidulafungin		16	38	15	**2**		**1**							69	95.8%	3	4.2%	3	4.2%
Micafungin				7	54	7	**3**				**1**			IE	IE	IE	IE	4	5.6%
Fluconazole											2	16	54	0	0	72	100%	ND	ND
Voriconazole					16	36	13	5	**1**		**1**			IE	IE	IE	IE	2	2.8%
***C. parapsilosis sensu stricto*** (*n* = 61)																			
Amphotericin B					1	18	41	1						61	100%	0	0	0	0
Anidulafungin						1	20	21	15	4				61	100%	0	0	0	0
Micafungin						1	2	27	30	**1**				60	98.4%	1	1.6%	1	1.6%
Fluconazole						1	34	20	5				**1**	60	98.%	1	1.6%	1	1.6%
Voriconazole			58	2			**1**							60	98.4%	1	1.6%	1	1.6%
***C. tropicalis*** (*n* = 75)																			
Amphotericin B					4	36	34	1						75	1	0	0	0	0
Anidulafungin	13	43	19											75	1	0	0	0	0
Micafungin	6	12	53	4										IE	IE	IE	IE	0	0
Fluconazole					4	27	25	13	**1**	**2**	**1**	**1**	**1**	70	93.3%	3	4.0%	6	8.0%
Voriconazole			66	3		**2**		**1**	**1**		**2**			69	92.0%	4	5.3%	6	8.0%
***C.* species** (*n* = 65)																			
Amphotericin B			1	3	17	25	13	6						43	100	0	0	ND	ND
Anidulafungin	6	7	17	10	6	8	3	7	1					ND	ND	ND	ND	ND	ND
Micafungin		4	10	23	6	10	12							ND	ND	ND	ND	ND	ND
Fluconazole					1	10	16	6	9	10	2	5	6	42	64.6%	13	20%	ND	ND
Voriconazole			38	5	13	5	2	2						ND	ND	ND	ND	ND	ND
**Other fungi**																			
Amphotericin B (*n* = 26)				2	4	9	4	6	1					25	96.2%	1	3.8%	ND	ND
Anidulafungin (*n* = 20)		2	1	3	1	2		1	1	2	7			ND	ND	ND	ND	ND	ND
Micafungin (*n* = 20)				3	3	2		2			10			ND	ND	ND	ND	ND	ND
Fluconazole (*n* = 25)								2	4	4	5	7	3	6	24.0%	15	60.0%	ND	ND
Voriconazole (*n* = 25)			5	6	4	5	2		1		1	1		ND	ND	ND	ND	ND	ND
**Overall** (*n* = 1439)																			
Amphotericin B (*n* = 1439)		6	32	63	280	682	336	39	1					1416	99.9%	1	0.001%	ND	ND
Anidulafungin (*n* = 1433)	643	293	294	93	10	14	25	31	17	6	7			ND	ND	ND	ND	ND	ND
Micafungin (*n* = 1433)	469	522	220	45	65	20	19	30	31	1	11			ND	ND	ND	ND	ND	ND
Fluconazole (*n* = 1438)					313	369	106	52	162	243	38	36	119	848	59.0%	159 **	11.1%	ND	ND
Voriconazole (*n* = 1438)			891	271	127	57	23	20	30	10	8	1		ND	ND	ND	ND	ND	ND

The abbreviation *n* indicates the number of isolates that were tested and had an MIC value; IE: Insufficient evidence; ND: not determined. Non-WT MICs are in bold and resistant underlined. MIC values outside the tested range are marked in grey. For other fungi, numbers of isolates tested for amphotericin B (*n* = 26), for anidualfungin and micafungin (*n* = 20), for fluconazole (*n* = 25), for voriconazole (*n* = 25). * Four isolates with MIC ≤0.004 mg/L and 33 isolates with MIC 0.008 mg/L. ** The stated number of 159 resistant isolates for fluconazole under ‘Overall’ is based on the individual-species-specific breakpoints. None of the tested *C. parapsilosis* isolates had an MIC value stated “>1” for anidulafungin or micafungin. Over time, several concentration ranges were used. Only uniform ranges are included and shown in the table. Not all isolates were tested for all antifungal agents if known to be intrinsically resistant.

**Table 4:** Identified *FKS* amino acid (AA) alterations. Unless stated otherwise, the detected alterations are known to be associated with echinocandin resistance.

Species (*n*)	Echinocandin Resistant (*n*)	*FKS* Alteration	Hotspot (HS) Location
Isolates with hotspot alterations			
*C. glabrata* (1)	Yes	F625S	Fks1
*C. glabrata* (2)	Yes	L630Q and S663F	Fks1and Fks2, respectively
*C. glabrata* (3)	Yes	F659S	Fks2
*C. glabrata* (1)	Yes	F659del	Fks2
*C. glabrata* (1)	Yes	L662W	Fks2
*C. glabrata* (3)	Yes	S663F	Fks2
*C. glabrata* (1)	Yes	S663P	Fks2
*C. krusei* (2)	Yes	S659S/F	Fks1
*C. krusei* (1)	Yes	S659F	Fks1
*C. dubliniensis* (1)	Yes	S645P	Fks1
Isolates with alterations outside the hotspots			
*C. krusei* (4)	Yes (2) No (2)	L701M *	Fks1; 38 AA after HS1
*C. albicans* (1)	Yes	P1354P/S **	Fks1; 3 AA before HS2
*C. lusitaniae* (1)	No	(H657Y/L1243F/ I1283V) **	Fks 1; 15 AA after HS1; 105 AA before HS2 and 65 AA before HS2

* L701M is located 38 AA after hotspot 1 and is not associated with echinocandin resistance. ** Unknown association to echinocandin resistance.

## Data Availability

Data are only available for research upon reasonable request to Statens Serum Institut and within the framework of the Danish data protection legislation.

## Supplementary Materials

The following are available online at https://www.mdpi.com/article/10.3390/jof7060491/s1. Table S1: Centre based data. Species distribution, blood culture systems and episode rates at the ten clinical microbiological departments in 2016 to 2018. Table S2: Number of patients related to age groups. Table S3: P-values for comparisons of episodes in relation to number of inhabitants in patient groups. Figure S1: Annual consumption of itraconazole and posaconazole.
