# Supplementary material for: Update 2016–2018 of the Nationwide Danish Fungaemia Surveillance Study: Epidemiologic Changes in a 15-Year Perspective
Source: J Fungi (Basel). 2021 Jun 19;7(6):491. doi: 10.3390/jof7060491 (PMC8235436; doi:10.3390/jof7060491)
Supplement: Supplementary file 1 [file jof-07-00491-s001.zip › jof-1254181-supplementary.pdf]

Journal of *Fungi*

MDPI

Supplementary Material

Update 2016–2018 of the Nationwide Danish Fungaemia Surveillance Study: Epidemiologic Changes in a 15-Year Perspective

Table S1. Centre based data. Species distribution, blood culture systems and episode rates at the ten clinical microbiological departments in 2016 to 2018.

Species	Rigshospitalet	Hvidovre	Herlev	Slagelse	Sønderborg	Esbjerg	Vejle	Århus	Ålborg	Odense
**Number of isolates**	215	175	163	177	21	26	60	258	149	210
**Species distribution**										
C. albicans	34.4%	40.6%	40.5%	39.5%	33.3%	34.6%	48.3%	48.4%	46.3%	43.8%
C. dubliniensis	10.2%	6.3%	3.7%	5.1%	0.0%	0.0%	3.3%	3.9%	2.7%	4.3%
C. glabrata	24.7%	26.9%	38.0%	36.2%	42.9%	57.7%	26.7%	28.7%	36.9%	34.3%
C. krusei	8.8%	6.9%	3.1%	4.0%	0.0%	0.0%	3.3%	6.2%	2.7%	3.8%
C. parapsilosis	6.5%	2.9%	5.5%	2.8%	4.8%	7.7%	0.0%	3.5%	6.0%	3.8%
C. tropicalis	4.7%	6.9%	3.7%	6.8%	0.0%	0.0%	13.3%	3.9%	2.7%	6.2%
Candida spp.	7.0%	7.4%	4.3%	4.0%	9.5%	0.0%	3.3%	3.9%	2.7%	2.4%
Other fungi	3.7%	2.3%	1.2%	1.7%	9.5%	0.0%	1.7%	1.6%	0.0%	1.4%
Blood culture systeme[1]	BT	BA*	BT**	BA	BA	BA (6 ms) BT (30 ms)	BA (18 ms) BT (18 ms)	BA	BT	BA
Proportion of patients < 50 years	30.8%	11.6%	3.9%	10.1%	5%	12%	9.6%	14.3%	13.8%	15.2%
Episodes (n)	207	174	162	169	21	26	55	250	139	199
**Incidences**										
/100.000 inhabitants	N/A	5.62	6.97	6.75	3.04	3.94	6.23	6.39	7.89	14.55
/1000 discharges	0.62	0.33	0.26	0.24	0.21	0.27	0.38	0.31	0.40	0.69
/10.000 bed days	1.69	1.17	0.93	0.98	0.77	0.85	1.09	1.07	0.59	1.94

[1] BA: BacT/ALERT, BT: BACTEC. * Selected departments used BACTEC (BT). ** Selected departments used BacT/ALERT (BA). ms: months. Rigshospitalet is a referral hospital.

Table S2. Number of patients related to age groups.

Age Group	Number of Patients	% of All
<1	17	1.3%
1–9	17	1.3%
10–19	15	1.1%
20–29	19	1.4%
30–39	34	2.6%
40–49	82	6.3%
50–59	179	13.7%
60–69	269	20.5%
70–79	423	32.3%
80–89	218	16.6%
90+	38	2.9%

Table S3. *p*-values for comparisons of episodes in relation to number of inhabitants in patient groups.

Males		Females	
**Age Group (Years)**	***p*-Value**	**Age Group (Years)**	***p*-Value**
<1	0.000	<1	0.002
1–9	0.000	1–9	0.000
10–19	0.000	10–19	0.000
20–29	0.000	20–29	0.000
30–39	0.000	30–39	0.000
40–49	0.000	40–49	0.000
50–59	0.000	50–59	0.000
60–69	0.000	60–69	0.000
70–79	0.006	70–79	0.018
80–89	0.100	80–89*	
90+*		90+	0.032

A poisson regression analysis was used. *Reference group in which number of episodes per 100,000 inhabitant was highest.

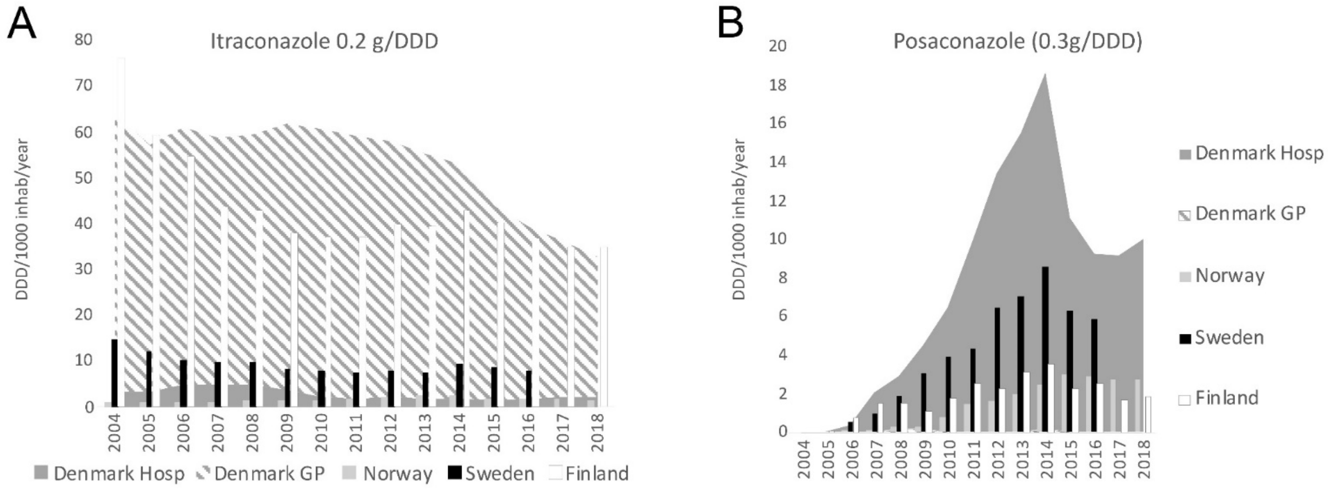

Figure S1. Annual consumption of itraconazole and posaconazole shown in DDDs per 1000 inhabitants per year in 2004 to 2018. For posaconazole, new formulations (iv and enterotablet; maintenance dose 300 mg/day as compared to 800 mg/day for the initial oral suspension) were introduced in 2014. In 2017, the DDD was changed from 0.8 g to 0.3 g.https://www.whocc.no/atc_ddd_alterations__cumulative/ddd_alterations/(Accessed on 17 June 2021) . This resulted in a higher DDD than previously measured, especially for the years prior to 2014 with exclusive use of oral suspension. Detailed data based on posaconazole formulations from 2004 to 2015 are shown in the surveillance by Astvad et al., and data from 2004 to 2015 have previously been published [10].
